# Multi locus sequence typing of *Chlamydiales*: clonal groupings within the obligate intracellular bacteria *Chlamydia trachomatis*

**DOI:** 10.1186/1471-2180-8-42

**Published:** 2008-02-28

**Authors:** Yvonne Pannekoek, Giovanna Morelli, Barica Kusecek, Servaas A Morré, Jacobus M Ossewaarde, Ankie A Langerak, Arie van der Ende

**Affiliations:** 1 Department of Medical Microbiology, Center for Infection and Immunity Amsterdam (CINIMA), Academic Medical Center, Amsterdam, The Netherlands; 2 Department of Molecular Biology, Max Planck Institute for Infection Biology, Berlin, Germany; 3 Department of Pathology, Laboratory of Immunogenetics, Section Immunogenetics of Infectious Diseases, & Department of Internal Medicine, Section Infectious Diseases, Free University Medical Center, Amsterdam, The Netherlands; 4 Department of Medical Microbiology and Infectious Diseases, Erasmus Medical Centre and Laboratory of Medical Microbiology, Medisch Centrum Rijnmond-Zuid, Rotterdam, The Netherlands

## Abstract

**Background:**

The obligate intracellular growing bacterium *Chlamydia trachomatis *causes diseases like trachoma, urogenital infection and lymphogranuloma venereum with severe morbidity. Several serovars and genotypes have been identified, but these could not be linked to clinical disease or outcome. The related *Chlamydophila pneumoniae*, of which no subtypes are recognized, causes respiratory infections worldwide. We developed a multi locus sequence typing (MLST) scheme to understand the population genetic structure and diversity of these species and to evaluate the association between genotype and disease.

**Results:**

A collection of 26 strains of *C. trachomatis *of different serovars and clinical presentation and 18 strains of *C. pneumoniae *were included in the study. For comparison, sequences of *C. abortus, C. psittaci*, *C. caviae*, *C. felis*, *C. pecorum *(*Chlamydophila*), *C. muridarum *(*Chlamydia*) and of *Candidatus protochlamydia *and *Simkania negevensis *were also included. Sequences of fragments (400 – 500 base pairs) from seven housekeeping genes (*enoA*, *fumC*, *gatA*, *gidA*, *hemN*, *hlfX*, *oppA*) were analysed. Analysis of allelic profiles by eBurst revealed three non-overlapping clonal complexes among the *C. trachomatis *strains, while the *C. pneumoniae *strains formed a single group. An UPGMA tree produced from the allelic profiles resulted in three groups of sequence types. The LGV strains grouped in a single cluster, while the urogenital strains were distributed over two separated groups, one consisted solely of strains with frequent occurring serovars (E, D and F). The distribution of the different serovars over the three groups was not consistent, suggesting exchange of serovar encoding *ompA *sequences. In one instance, exchange of *fumC *sequences between strains of different groups was observed. Cluster analyses of concatenated sequences of the Chlamydophila and Chlamydia species together with those of *Candidatus Protochlamydia amoebophila *and *Simkania negevensis *resulted in a tree identical to that obtained with 23S RNA gene sequences.

**Conclusion:**

These data show that *C. trachomatis *and *C. pneumoniae *are highly uniform. The difference in genetic diversity between *C. trachomatis *and *C. pneumoniae *is in concordance with a later assimilation to the human host of the latter. Our data supports the taxonomy of the order of *Chlamydiales*.

## Background

*Chlamydia trachomatis *is the world's leading cause of preventable blindness. Also, *C. trachomatis *is considered the world's most common sexually transmitted bacterial pathogen. Many urogenital infections remain unnoticed, constituting a large reservoir of untreated individuals, a continuous threat for transmission of this pathogen. When not treated in time, infection with *C. trachomatis *can lead to infertility in women. *C. trachomatis *strains are discriminated by serotyping based on the antigenic difference between the major outer membrane proteins (MOMP). Nineteen serovars have been described: A, B, Ba, C (mainly seen among isolates from trachoma infections) D, Da, E, F, G, Ga, H, I, Ia, J, Ja, K, (urogenital infections) and L1, L2, L2a and L3 causing lymphogranuloma venereum (LGV). Among urogenital infections, serovars D – F are most frequently found [1]. However, serotyping is laborious, needing culture and a large panel of antibodies [2,3]. To overcome these drawbacks a PCR based RFLP of *ompA *was developed for the identification of genotypes corresponding to serovars [4-6]. Using this method genotypes were categorised into three geno-groups: the B group (B, E, D, Da, L1, L2, L2a), the C group (C, A, H, I, Ia, J, K, L3) and the intermediate group (F, G, Ga). Except for an immunological relationship between members of a group, the biological relevance of the geno-groups remains obscure.

*Chlamydophila pneumoniae *is a common cause of community-acquired pneumonia, bronchitis, pharyngitis and sinusitis [7]. Although *C. pneumoniae *often causes mild or subclinical infections, its persistence in the host can lead to the establishment of chronic pathologies and an increasing number of reports indicate an association between persistent *C. pneumoniae *infections and arteriosclerosis [8] or coronary heart diseases [9,10]. A robust typing scheme for *C. pneumoniae *is lacking.

Together with *C. trachomatis, C. pneumoniae *belongs to the family of *Chlamydiaceae *in the order of *Chlamydiales*. Based on phylogenetic analyses of 16S and 23S rRNA gene sequences, *C. trachomatis, Chlamydia suis *and *Chlamydia muridarum *all belong to the genus *Chlamydia*, while *C. pneumoniae*, *Chlamydophila psittaci*, *Chlamydophila pecorum*, *Chlamydophila felis*, *Chlamydophila abortus*, and *Chlamydophila caviae *all belong to the family of *Chlamydophila *[11-13]. Other family members of the order of *Chlamydiales *are *Parachlamydiaceae *and *Simkaniaceae*.

Currently, the typing scheme for *C. trachomatis *is based on epitopes in the major outer membrane protein (MOMP). Variants of this protein are subjected to selection and isolates of the same serovar may not be closely related [14,15]. Here we present an MLST typing scheme using gene segment sequences of seven housekeeping genes. These genes were selected using the criteria that they are widely separated on the chromosome and not adjacent to putative outer membrane, secreted, or hypothetical proteins that might be under diversifying selection. In addition, each locus has a similar extent of nucleotide substitutions to ensure consistency [16]. The results identified three sub-groupings within *C. trachomatis*, but no subdivision within *C. pneumoniae*. A phylogenetic tree based on the concatenated sequences of six of the housekeeping gene fragments is consistent with a tree based on 16S and 23S rRNA gene sequences.

## Results

### MLST of *C. trachomatis *and *C. pneumoniae*

Analogous to the MLST schemes of e.g. *Neisseria meningitidis *[17] and *Streptococcus pneumoniae *[18] fragments of seven housekeeping genes scattered around the chromosome of *C. trachomatis *and *C. pneumoniae *were obtained (Tables 1 and 2). The gene order on the chromosome of both species is identical. None of the sequences of the seven different loci among the *C. trachomatis *strains contained gaps after alignment.

**Table 1:** Properties of gene fragments sequenced, 26 *C. trachomatis *strains

gene	locus tag	position in genome of Ct A/HAR-13	Length (bp)	No. of synonymous substitutions	d_S_^a^	No. of non-synonymous substitutions	d_N_^a^
*gatA*	CTA_0003	2759 – 3183	425	2	0.00409	0	0.00000
*oppA*_3	CTA_0216	224745 – 225217	473	3	0.00209	3	0.00248
*hflX*	CTA_0413	435569 – 435135	435	2	0.00663	1	0.00151
*gidA*	CTA_0546	580102 – 580575	474	1	0.00235	3	0.00205
*enoA*	CTA_0637	665021 – 665401	381	2	0.00562	1	0.00094
*hemN*	CTA_0812	871011 – 870580	432	0	0.00000	1	0.00082
*fumC*	CTA_0932	1007488 – 1007952	465	0	0.00000	4	0.00217

Total			3085	10	0.00294	13	0.00147

^a^Jukes & Cantor corrected

**Table 2:** Properties of gene fragments sequenced, 18 *C. pneumoniae *strains.

gene	locus tag	position in genome of Cp CWL029	Length (bp)	No. of synonymous substitutions	d_S_^a^	No. of non-synonymous substitutions	d_N_^a^
*gatA*	CPn0003	952 – 1376	425	0	0.00000	0	0
*oppA_1*	CPn0195	234253 – 234746	494	0	0.00000	0	0
*hflX*	CPn0478	557187 – 556753	435	0	0.00000	0	0
*gidA*	CPn0617	710937 – 711410	474	1	0.00373	0	0
*enoA*	CPn0800	906070 – 905690	381	1	0.00124	1	0.00147
*hemN*	CPn0889	1016340 – 1015909	432	0	0.00000	0	0
*fumC*	CPn1013	1162452 – 1162916	465	0	0.00000	0	0

Total			3106	2	0.00073	1	0.00018

^a^Jukes & Cantor corrected

Variation among the sequences of the seven loci was very limited. In *C. trachomatis *the highest number (three) of synonymous substitutions was seen in *oppA *while the highest number (four) of non-synonymous substitutions was seen in *fumC*. Analogous to other MLST schemes, we assigned allele numbers to each unique allele sequence for each house-keeping gene [16]. The number of alleles per locus varied between two and six. Most of the alleles were seen more than once. However, among the *oppA *alleles four unique alleles were found while among the *gidA *and *enoA *sequences one unique allele was observed.

**Figure 1:**
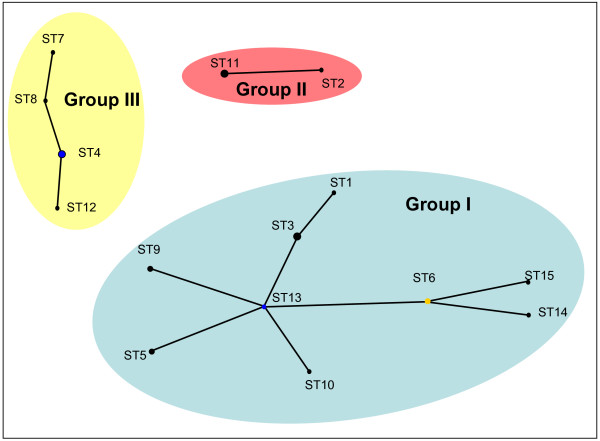
**Clonal groupings among C. trachomatis strains**. Allelic profiles were analysed by eBurst and groups were defined as sets of related strains sharing identical alleles at six of the seven loci loci with at least one other member of the group. Blue dot in group I indicate the putative founder, yellow dot that of a subgroup.

**Figure 2:**
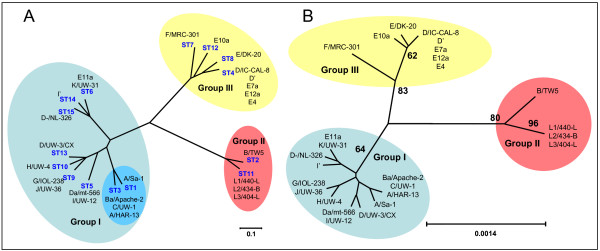
**Phylogenetic analyses of seven housekeeping gene fragments of C. trachomatis strains**. A) The tree was constructed using UPGMA algorithm in SplitsTree4 using MLST allelic profiles. Distance matrix was obtained from allelic profiles using the SplitsTree program. B) UPGMA cluster analyses, with Jukes-Cantor correction, using concatenated sequences. Bold numbers indicate bootstrap values over 50%. Horizontal lines are scale for genetic distance.

**Figure 3:**
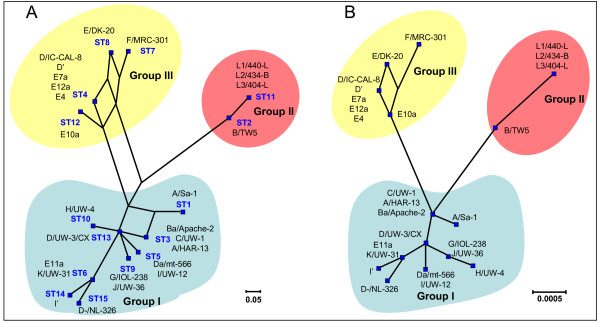
**SplitsTree decomposition analyses of MLST data of C. trachomatis strains**. A) SplitsTree decomposition network was obtained using distance matrix obtained from allelelic profiles in SplitsTree4. B) SplitsTree decomposition network was obtained using distance matrix obtained using concatenated sequences.

For each isolate, the alleles at each of the seven loci define the allelic profile or sequence type (ST). Among the 26 *C. trachomatis *strains 15 ST's could be assigned. Analysis by eBurst revealed three non-overlapping groups or clonal complexes, consisting of related strains sharing identical alleles at six of the seven loci with at least one other member of the group (Figure 1). An UPGMA cluster analysis showed the same groups (Figure 2A). SplitsTree decomposition demonstrated that alternative routes of descent in the tree resulted in the same groupings (Figure 3). An UPGMA cluster analyses of the concatenated sequences of the seven gene fragments yielded the same groupings as when allelic profiles were used (Figure 2B), while SplitsTree decomposition analysis yielded a more simpler network but with the same groupings as with the distances matrix of allelic profiles.

Group I, with ST13 as the putative founder, defined as the ST with the most single locus variants, and ST6 as the founder of a subgroup consisted of *C. trachomatis *strains isolated from patients with urogenital infection (serovars D to K) as well as trachoma infections (serovars A, Ba C). The latter formed a separate branch in the UPGMA cluster analyses. Group II comprises the LGV strains (serovar L) and strain B/TW-5 (serovar B). Group III is formed by all, except one, serovar E strains and one serovar F and two serovar D strains.

Sequence variation among *C. pneumoniae *was far less than among *C. trachomatis *(Table 1). Substitutions were only seen among the sequences of *gidA *(1 synonymous) and *enoA *(1 synonymous and 1 non-synonymous). This means that all 16 strains shared identical alleles at least five of the seven loci, i.e. *C. pneumoniae *appeared to be highly uniform. In addition, none of the alleles in *C. trachomatis *and *C. pneumoniae *are the same.

### Recombination in *C. trachomatis*

**Figure 4:**
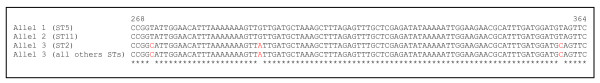
**Nucleotide substitutions among the three fumC alleles of C. trachomatis**. Sequences between position 268 and 364 with the three nucleotide substitutions are shown. Allele 1 (ST5) has a nucleotide substitution at position 137 (not shown).

In group II, the allelic profile of ST2 (B/TW-5) differs from that of ST11 (serovar L strains) at one locus (Table 3). It shares the *fumC *allele with the majority of the strains in group I and II, indicating exchanges of the *fumC *sequences between a strain with genotype ST11 and a strain with genotype other than ST11 or ST5 (with *fumC *allele different from that of ST1 and all other ST's; Table 3), resulting the B/TW-5 strain with genotype ST2. The difference in *fumC *sequences are three substitutions in an 89 bp region, albeit that all three appear to be non-synonymous (Figure 4).

**Table 3:** Alelelic profiles of Sequence Types (ST) among *C. trachomatis*

	**Allele no. of housekeeping locus**							
		
**ST**	***gatA***	***oppA***	***hflX***	***gidA***	***enoA***	***hemN***	***fumC***	**clonal group**
ST1	3	3	4	5	1	2	3	I
ST2	1	3	3	3	2	2	3	II
ST3	3	3	4	5	3	2	3	I
ST4	3	1	1	2	4	2	3	III
ST5	3	3	2	5	3	2	1	I
ST6	3	3	2	5	3	1	3	I
ST7	2	2	1	2	4	2	3	III
ST8	2	1	1	2	4	2	3	III
ST9	3	3	2	4	3	2	3	I
ST10	3	3	2	1	3	2	3	I
ST11	1	3	3	3	2	2	2	II
ST12	3	4	1	2	4	2	3	III
ST13	3	3	2	5	3	2	3	I
ST14	3	6	2	5	3	1	3	I
ST15	3	5	2	5	3	1	3	I

In addition, while ST11 strains are serovar L, ST 2 is serovar B, indication exchange of *ompA *(encoding MOMP, defining the serovar type) sequences between a serovar B strain and a serovar L strain. Other indications of recombination between different *C. trachomatis *genotypes and exchange of *ompA *sequences might be inferred from the position of the only serovar E (serovar E11A, ST6) in group I, while all other serovar E strains cluster in group III.

### MLSA based phylogeny of Chlamydiales

The *oppA *sequences of *C. pneumoniae *contained several indels when compared to the sequences of *C. trachomatis *and other species of *Chlamydiales*. All genomes of the *Chlamydiales *contain multiple copies of *oppA *genes. In each genome, these copies are highly homologous, but vary between the different species, making selection of the right *oppA *copy from these genome sequences indecisive.

**Figure 5:**
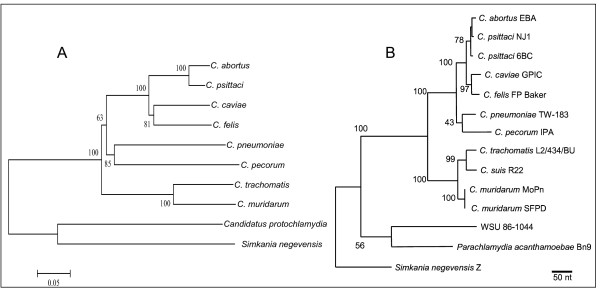
**Phylogenetic analyses of concatenated sequences of 6 housekeeping gene fragments**. A) Concatenated sequences of six housekeeping gene fragments were aligned and analysed in MEGA 3.1. Phylogenetic tree was constructed using the Neighbour-Joining algorithm with Kimura-2 parameter. Bootstrap test was for 1000 repetitions. Bold numbers indicate bootstrap values over 50%. Horizontal lines are scale for genetic distance. B) Phylogenetic tree based 23S rRNA gene sequences (Adapted from Everett et al [10]).

An insert was observed in the *enoA *sequences of *Candidatus protochlamydia *and *Simkania negevensis*. Also, the *hemN *sequences of these strains contained small indels as compared to the *hemN *sequences of the other members of the *Chlamydiales*. Small indels were also observed among the *hlfX *sequences of *C. abortus*, *C. caviae*, *C. felis*, *C. psittaci*, *Candidatus protochlamydia *and *Simkania negevensis*. Recently, multilocus sequence analysis (MLSA) was introduced to study relatedness of closely related species [19,20]. In this analysis the sequences of multi locus housekeeping fragments are concatenated and used in cluster analysis. Phylogenetic analysis of the *Chlamydiales *by Neighbour-Joining method of the aligned concatenated sequences of the housekeeping gene fragments, except that of *oppA*, resulted in a tree (Fig 5A) comparable to that obtained with 16S rRNA gene and 23S rRNA gene (Fig 5B) sequences [11].

## Discussion

To assess the population structure of *C. trachomatis *and *C. pneumoniae *sequences of fragments of seven housekeeping genes, obtained from 26 *C. trachomatis *strains and 18 *C. pneumoniae *strains, were analysed. *C. pneumoniae *appeared to be highly uniform. Among the *C. trachomatis *strains three very coherent clonal complexes were observed, consisting of strains sharing identical alleles of at least 6 of the 7 loci with one other member of the group. *C. pneumoniae *appeared to be highly uniform.

Recently, an MLST scheme has been published, in which five target regions were selected based on their relatively high variability as compared to the rest of the genome and analysed. In addition, these targets were not widely separated on the genome [21]. That typing scheme was intended to be highly discriminatory and to be applied in contact tracing.

In the present study, 7 housekeeping genes were chosen, which were widely separated on the chromosome and not adjacent to putative outer membrane, secreted, or hypothetical proteins that might be under diversifying selection. In addition, each locus has a similar level of variation in terms of nucleotide substitutions to ensure consistency [16]. Fifteen sequence types were found among 26 *C. trachomatis *isolates (0.6 STs per isolate). Many organisms show more diversity, i.e. more sequence types per isolate, but a significant number of organisms shows comparable or less sequence types per isolate [16]. In these organisms MLST has been applied for strain characterization and epidemiological surveillance (*e.g. Listeria monocytogenes*, 29 STs among 62 isolates; *Acinetobacter baumannii*, 20 STs among 49 isolates), population structure analyses (e.g. *Porphyromonas gingivalis*, 50 STs in 59 isolates) and evolutionary analyses studies (e.g. *Batrachochytrium dendrobatidis*, 10 STs in 35 isolates) [16]. The here presented MLST scheme for *C. trachomatis *may be similarly used. Our results show three clonal complexes among *C. trachomatis *of which one is associated with LGV.

A phylogeny tree based on the concatenated sequences of 6 loci resulted in a tree consistent with that of obtained when 16S rRNA and 23S rRNA genes were used in the phylogeny analyses [11]. This approach, using the concatenated sequences to study the relationships among strains of similar species was recently termed multilocus sequence analysis (MLSA) [19] and has successfully been applied to other species [20].

*C. trachomatis *show limited variation; the average number of synonymous substitutions in *C. trachomatis *is in the same order as that in *Yersinia pseudotuberculosis *[22-24]. In contrast, the average number of synonymous substitutions in *C. pneumoniae *is even smaller, comparable to that in *Vibrio sonnei *and *Yersinia pestis*, but larger than in *Mycobacterium tuberculosis *[23-25]. This may suggest that both species *C. trachomatis *and *C. pneumoniae *are evolutionarily young or recently past severe bottle necks [26].

Three clonal complexes were seen among the 26 strains of *C. trachomatis*; each group includes isolates that differ at only one locus from at least one other isolates within the group. Singeltons, differing at two or more loci from all other isolates were not observed. Our data provided some evidence of recombination, e.g. exchange of the MOMP (serovar determining) encoding *ompA *sequences and of *fumC *sequences. Discongruence between *ompA *and the main part of the genome has also been observed by Gomes and colleagues and Brunelle and Sensabaugh [15,27]. In addition, earlier reports of mosaic *ompA *gene structures indicated that *ompA *or parts of *ompA *do exchange between *C. trachomatis *strains [28-30].

Brunelle and Sensabaugh observed recombination in *ompA *genes, *pmpE *genes and *pmpH *genes, but not in the remainder of the genome [15]. Recently published data by Gomes and colleagues suggested frequent recombination in *C. trachomatis*, albeit that this recombination occurred at hotspot near or in *ompA *and *pmp *genes [14]. Here we demonstrated in at least one instance recombination in or near the housekeeping *fumC*. The allelic profile of ST2 was identical to that of ST11 with the exception of the *fumC *allele. The *fumC *allele of ST2 was identical to that of the majority of the other *C. trachomatis *strains, while that of the ST11 strains differed at three positions within 87 nucleotides, suggesting uptake and recombination of (a part of) *fumC *sequence by an ST11 genotype *C. trachomatis *resulting in ST2 genotype. It is unlikely that *pmp *or *ompA *sequences are involved in the exchange of *fumC *sequences, since the nearest *pmp *genes are 54 Kbp upstream (*pmpD*, cta0884) and 23 Kbp upstream (*pmpE*, cta0949) of *fumC*.

The three clonal complexes or groups are partly associated with tissue tropism. All LGV causing strains group together in group II. The urogenital strains and ocular strains are distributed over two groups, albeit that the ocular strains group together with the less frequent occurring urogenital strains (serovar H to K). In addition, the trachoma strains form a separated branch within group I. The more frequently occurring urogenital strains formed the separate group III. High frequency occurring genotypes may be linked with symptomatic infection, but in a study among woman with urogenital *C. trachomatis *infections serovar E and F strains were equally isolated from patients with symptoms and from patients without symptoms [31]. Hence, host factors may determine disease outcome. Alternatively, the high frequency occurring genotypes may be associated with higher transmission rates.

## Conclusion

The *C. pneumoniae *population is highly uniform, while that of *C. trachomatis *shows three clonal complexes based on an MLST scheme of 7 housekeeping genes. More clonal groups may be identified when more strains will be analysed with this scheme. The difference in genetic diversity between *C. trachomatis *and *C. pneumoniae *is in concordance with a later assimilation to the human host of the latter.

## Methods

### Strain collection

Twenty-four *C. trachomatis *strains were used, consisting of reference strains: (A/Sa-1, H/UW-4, I', D-, Ba/Apache-2, C/UW-1, Da/mt-566, I/UW-12, K/UW-31, G/IOL-238, J/UW-36, L1/440-L, L2/434-B, L3/404-L, B/TW5, D/IC-CAL-8, D', F/MRC-301 and E/DK-20 [32-34] and additional new isolates from patients, E4a, E7a, E10a, E11a, and E12a [see additional file 1]. The serovar of these isolates was confirmed by RFLP [32,35]. We also tested 14 reference strains of *C. pneumoniae *(CM-1, IOL-207, PS-32, AR-338, BAL-16, CWL-011, CWL-50, GRO-21, H-12, K-7, NWL-1, UZG-1, 2023, 2043) [33] [see additional file 1]. In addition, we also analyzed *C. trachomatis *strains D/UW-3/CX (Accession no. AE001273) [36], A/HAR-13 (Accession no. NC_007429) [37] and *C. pneumoniae *strain CWL-029 (Accession no. NC_000922) [38], AR-39 (Accession no. AE002161) [39], TW-183 (Accession no. AE017160) and J138 (Accession no. BA000008) [40] whose complete genome sequences are available in the database. The collection of *C. trachomatis *and *C. pneumoniae *strains represents all known serovars and were from patients with various disease outcomes (see additional file 1). In addition, the corresponding sequences from *C. muridarum *Nigg. (Accession no. AE002160) (Chlamydia) [39] and of *C. abortus *S26/3 (Accession no. NC_004552) [41], *C. caviae *GPIC (Accession no. AE015925) [42], *C. felis *Fe/C-56 (Accession no. AP006861) [43] and of *Candidatus protochlamydia amoebophila *UWE25 (Accession no. BX908798) (*Parachlamydiaceae*) [22] were obtained from publicly accessible databases. The corresponding sequences from *C. pecorum *E58 (McNutt), *C. psittaci *6BC and *Simkania negevensis *(Simkaniaceae) were obtained from TIGR's unfinished genomes database.

### DNA, genes, PCR products and sequences

DNA was extracted from elementary bodies from cultures of *C. trachomatis *or *C. pneumoniae *according to Boom *et al *[44]. Fragments of 7 genes, i.e. *gatA*, *oppA*3, *hflX*, *gidA*, *enoA*, *hemN *and *fumC *encoding aspartyl/glutamyl-tRNA amidotransferase subunit A, oligopeptide-binding protein, GTP-binding protein, tRNA (uracil-5-)-methyltransferase, enolase, coproporphyrinogen III oxidase and fumarate hydratase, respectively were amplified using the oligonucleotide primers shown in Table 4. Amplification primers were designed based on the genome sequence of A/HAR-13 to yield amplicons that were short enough to obtain complete double stranded sequences in two single sequence runs. Each sequence run was performed from a different PCR amplicon and sequence traces were obtained with ABI Big-dyes and an ABI 3730 sequencer.

**Table 4:** Oligonucleotide primers

**Target gene**	**Forward primer**	**Reversed primer**
*gatA*	GCTTTAGAATTARSARAWGCT	GATCCTCCGGTATCYGATCC
*oppA*_3	ATGCGCAAGATATCAGTGGG	AAAGCTCCRSTWGMTATMGGWAG
*hflX*	GCTTCTARAGTACTTTTAAATG	TATTTRGAAATYTTTKCSAGYCG
*gidA*	GGAGTCWCTACWAAAGAAGG	TCGTAYTGYACATCRAAAGG
*enoA*	CCTATGATGAATCTKATCAATGG	TCTTCTTCRGCWAGMCCATCT
*hemN*	AGATCTTCTTCWGGRGGWAGAGA	TTCYTTCAKAACSTAGGTTTT
*fumC*	ATTAAAAAATGTGCTGCT	CCTTCAGGAACATTYAACCC

R = A or G; S = G or C; W = A or T; Y = C or T; M = A or C; K = G or T;

### Phylogenetic and other analyses

The number of synonymous and non-synonymous substitutions per site was determined using DnaSP 4.0 [45]. For *C. trachomatis*, unique sequences were assigned allele numbers using the Non-redundant databases (NRDB) program [46]. Allele profile data were analysed in eBurst to define clonal complexes or groups [47,48]. Groups were defined as sets of related strains containing pairs of strains that share at least six identical alleles at the seven loci.

A distance matrix in Nexus format was generated from the set of allelic profiles using SplitsTree [46]. This file was then used for phylogenetic analyses in SplitsTree 4.0 [49], both by generating an UPGMA tree and by SplitsTree decomposition analyses. Decomposition analysis depicts all the shortest pathways linking sequences, including those that produce an interconnected network.

Phylogenetic evolutionary analyses of the sequences of the different members of *Chlamydiales *were conducted using MEGA version 3.1 [50].

## Authors' contributions

YP participated in the design and coordination of the study and helped to draft the manuscript. GM participated in the design of the study and carried out the sequencing. BK participated in the design of the study and carried out the sequencing. SAM participated in the design of the study helped to draft the manuscript. JMO participated in the design and coordination of the study. AAL carried out sequencing. AvdE participated in the design and coordination of the study, did the analyses and interpretation of the sequence data and helped to draft the manuscript. All authors read and approved the final manuscript.

## Supplementary Material

Additional file 1 List of *Chlamydia trachomatis *en *Chlamydophila pneumoniae *strains. Table listing the strains used in this study. Click here for file
