# Supplementary material for: Multi locus sequence typing of Chlamydiales: clonal groupings within the obligate intracellular bacteria Chlamydia trachomatis
Source: BMC Microbiol. 2008 Feb 28;8:42. doi: 10.1186/1471-2180-8-42 (PMC2268939; doi:10.1186/1471-2180-8-42)
Supplement: Additional file 1 — List of Chlamydia trachomatis en Chlamydophila pneumoniae strains. Table listing the strains used in this study. [file 1471-2180-8-42-S1.doc]

List of *Chlamydia trachomatis* en *Chlamydophila pneumoniae* strains

| **Species** | **Isolate** | **Reference** | **Host** | **Clinical manifestation** | **Serovar** | **ST** | ***gatA*** | ***oppA*** | ***hflX*** | ***gidA*** | ***enoA*** | ***hemN*** | ***fumC*** | **group** |
| --- | --- | --- | --- | --- | --- | --- | --- | --- | --- | --- | --- | --- | --- | --- |
| *C. trachomatis* | A/HAR-13 | 37 | Human | trachoma | A | 3 | 3 | 3 | 4 | 5 | 3 | 2 | 3 | I |
|  | A/Sa-1 | 32-34 | Human | Trachoma | A | 1 | 3 | 3 | 4 | 5 | 1 | 2 | 3 | I |
|  | B/TW-5 | 32-34 | Human | Trachoma | B | 2 | 1 | 3 | 3 | 3 | 2 | 2 | 3 | II |
|  | Ba/Apache-2 | 32-34 | Human | Trachoma | B | 3 | 3 | 3 | 4 | 5 | 3 | 2 | 3 | I |
|  | C/UW-1 | 32-34 | Human | Trachoma | C | 3 | 3 | 3 | 4 | 5 | 3 | 2 | 3 | I |
|  | D/IC-CAL-8 | 32-34 | Human | Urogenital infection | D | 4 | 3 | 1 | 1 | 2 | 4 | 2 | 3 | III |
|  | Da/MT-566 | 32-34 | Human | Urogenital infection | D | 5 | 3 | 3 | 2 | 5 | 3 | 2 | 1 | I |
|  | D-/NL-326 | 32-34 | Human | Urogenital infection | D | 15 | 3 | 5 | 2 | 5 | 3 | 1 | 3 | I |
|  | D' | 32-34 | Human | Urogenital infection | D | 4 | 3 | 1 | 1 | 2 | 4 | 2 | 3 | III |
|  | D/UW-3/CX | 36 | Human | Urogenital infection | D | 13 | 3 | 3 | 2 | 5 | 3 | 2 | 3 | I |
|  | E/DK-20 | 32-34 | Human | Urogenital infection | E | 8 | 2 | 1 | 1 | 2 | 4 | 2 | 3 | III |
|  | E7a | - | Human | Urogenital infection | E | 4 | 3 | 1 | 1 | 2 | 4 | 2 | 3 | III |
|  | E10a | - | Human | Urogenital infection | E | 12 | 3 | 4 | 1 | 2 | 4 | 2 | 3 | III |
|  | E4a | - | Human | Urogenital infection | E | 4 | 3 | 1 | 1 | 2 | 4 | 2 | 3 | III |
|  | E11A | - | Human | Urogenital infection | E | 6 | 3 | 3 | 2 | 5 | 3 | 1 | 3 | I |
|  | F/MRC-301 | 32-34 | Human | Urogenital infection | F | 7 | 2 | 2 | 1 | 2 | 4 | 2 | 3 | III |
|  | G/IOL-238 | 32-34 | Human | Urogenital infection | G | 9 | 3 | 3 | 2 | 4 | 3 | 2 | 3 | I |
|  | H/UW-4 | 32-34 | Human | Urogenital infection | H | 10 | 3 | 3 | 2 | 1 | 3 | 2 | 3 | I |
|  | I/UW-12 | 32-34 | Human | Urogenital infection | I | 5 | 3 | 3 | 2 | 5 | 3 | 2 | 1 | I |
|  | I' | 32-34 | Human | Urogenital infection | I | 14 | 3 | 6 | 2 | 5 | 3 | 1 | 3 | I |
|  | J/UW-36 | 32-34 | Human | Urogenital infection | J | 9 | 3 | 3 | 2 | 4 | 3 | 2 | 3 | I |
|  | K/UW-31 | 32-34 | Human | Urogenital infection | K | 6 | 3 | 3 | 2 | 5 | 3 | 1 | 3 | I |
|  | L1/440-L | 32-34 | Human | Urogenital infection | L | 11 | 1 | 3 | 3 | 3 | 2 | 2 | 2 | II |
|  | L2/434-B | 32-34 | Human | Urogenital infection | L | 11 | 1 | 3 | 3 | 3 | 2 | 2 | 2 | II |
|  | L3/404-L | 32-34 | Human | Urogenital infection | L | 11 | 1 | 3 | 3 | 3 | 2 | 2 | 2 | II |
| *C. pneumoniae* | AR-39 | 39 | Human | Respiratory tract infection |  |  |  |  |  |  |  |  |  |  |
|  | AR-338 | 33 | Human | Respiratory tract infection |  |  |  |  |  |  |  |  |  |  |
|  | BAL-16 | 33 | Human | Bronchoalveolar lavage, patient with AIDS |  |  |  |  |  |  |  |  |  |  |
|  | CM-1 | 33 | Human | Pneumonia |  |  |  |  |  |  |  |  |  |  |
|  | CWL-011 | 33 | Human | Pneumonia |  |  |  |  |  |  |  |  |  |  |
|  | CWL-029 | 38 | Human | Pneumonia |  |  |  |  |  |  |  |  |  |  |
|  | CWL-050 | 33 | Human | Pneumonia |  |  |  |  |  |  |  |  |  |  |
|  | GRO-21 | 33 | Human | Pneumonia |  |  |  |  |  |  |  |  |  |  |
|  | H-12 | 33 | Human | Respiratory tract infection |  |  |  |  |  |  |  |  |  |  |
|  | IOL-207 | 33 | Human | Conjunctivitis |  |  |  |  |  |  |  |  |  |  |
|  | J138 | 40 | Human | Acute bronchitis |  |  |  |  |  |  |  |  |  |  |
|  | K-7 | 33 | Human | Respiratory tract infection |  |  |  |  |  |  |  |  |  |  |
|  | NWL-1 | 33 | Human | Pneumonia |  |  |  |  |  |  |  |  |  |  |
|  | TW-183 | 40 | Human | Conjunctivitis |  |  |  |  |  |  |  |  |  |  |
|  | UZG-1 | 33 | Human | Pneumonia |  |  |  |  |  |  |  |  |  |  |
|  | 2023 | 33 | Human | Respiratory tract infection |  |  |  |  |  |  |  |  |  |  |
|  | 2043 | 33 | Human | Pneumonia |  |  |  |  |  |  |  |  |  |  |
| *C. muridarum* | Nigg II | 39 | Mouse | Pneumonitis |  |  |  |  |  |  |  |  |  |  |
| *C. caviae* | GPIC | 42 | Caviae | conjunctivitis |  |  |  |  |  |  |  |  |  |  |
| *C. felis* | Fe/C-56 | 43 | Cat | Conjunctivis |  |  |  |  |  |  |  |  |  |  |
| *C. abortus* | S26/3 | 41 | Sheep | Abortion |  |  |  |  |  |  |  |  |  |  |
| *C. psittaci* | 6BC | TIGR | Parakeet | Psittacosis |  |  |  |  |  |  |  |  |  |  |
| *C. pecorum* | E58 (McNutt) | TIGR | Calf | Encephalitis |  |  |  |  |  |  |  |  |  |  |
| *Candidatus protochlamydia amoebophila* | UWE25 | 22 | Acanthamoeba castellanii |  |  |  |  |  |  |  |  |  |  |  |
| *Simkania negevensis* |  | TIGR | cell culture contaminant |  |  |  |  |  |  |  |  |  |  |  |
